# Incorporating representation learning and multihead attention to improve biomedical cross-sentence n-ary relation extraction

**DOI:** 10.1186/s12859-020-03629-9

**Published:** 2020-07-16

**Authors:** Di Zhao, Jian Wang, Yijia Zhang, Xin Wang, Hongfei Lin, Zhihao Yang

**Affiliations:** School of Computer Science and Technology, Dalian University of Technology, Dalian, China

**Keywords:** Biomedical n-ary relation, Multihead attention, Representation learning

## Abstract

**Background:**

Most biomedical information extraction focuses on binary relations within single sentences. However, extracting n-ary relations that span multiple sentences is in huge demand. At present, in the cross-sentence n-ary relation extraction task, the mainstream method not only relies heavily on syntactic parsing but also ignores prior knowledge.

**Results:**

In this paper, we propose a novel cross-sentence n-ary relation extraction method that utilizes the multihead attention and knowledge representation that is learned from the knowledge graph. Our model is built on self-attention, which can directly capture the relations between two words regardless of their syntactic relation. In addition, our method makes use of entity and relation information from the knowledge base to impose assistance while predicting the relation. Experiments on n-ary relation extraction show that combining context and knowledge representations can significantly improve the n-ary relation extraction performance. Meanwhile, we achieve comparable results with state-of-the-art methods.

**Conclusions:**

We explored a novel method for cross-sentence n-ary relation extraction. Unlike previous approaches, our methods operate directly on the sequence and learn how to model the internal structures of sentences. In addition, we introduce the knowledge representations learned from the knowledge graph into the cross-sentence n-ary relation extraction. Experiments based on knowledge representation learning show that entities and relations can be extracted in the knowledge graph, and coding this knowledge can provide consistent benefits.

## Background

The current tasks of biomedical relation extraction mainly focus on the extraction of binary relations in single sentences, such as protein-protein interaction (PPI), chemical-protein interaction (CPI) and drug-drug interaction (DDI) [1–3]. It is crucial for biomedical relation extraction to automatically construct a knowledge graph, which supports a variety of downstream natural language processing (NLP) tasks such as drug discovery [4]. An obvious problem is that as the biomedical literature continues to grow, there is a large number of biomedical entities whose binary relations exist not only in a single sentence but also in cross-sentences. In addition, the relations between entities are not merely a binary relation but may also be an n-ary relation. Consider the following example: the relations between drugs, genes and mutations. “*The deletion mutation on exon 19 of the****EGFR****gene was present in 16 patients, while the****L858E****point mutation on exon 21 was noted in 10. All patients were treated with****gefitinib****and showed a partial response.*”. The message conveyed by the two sentences is that there is a reaction between the three bold entities. As the biomedical literature contains a wealth of drug-gene-mutation relations, how to quickly and accurately identify the drug-gene-mutation relations is particularly important in the treatment of precision medicine [5].

Biomedical binary relation extraction is mainly divided into a rule-based method and a machine learning-based method [6]. The rule-based approach primarily uses the syntactic rules designed by linguists to extract relations between entities from documents. As the length of cross-sentence documents grows, the use of artificially designed language rules becomes complex and works inefficiently [7]. Neural networks are dominant in machine learning-based approaches. Neural networks do not require artificial design features and perform very well. The main methods are convolutional neural networks (CNNs), recurrent neural networks (RNNs), and their variants [8, 9]. CNN learns sequence local features through convolution kernels. RNN is a linear chain neural network that is ideal for processing sequence features. Compared with CNN, most biomedical relation extraction methods use RNN as the main framework. However, RNN also has certain limitations. As the sequences grow in length, a single memory unit requires powerful storage capabilities to preserve the complete information of long sequences. Additionally, the limitation is that RNN has difficulty processing tree structure documents, which ignores word dependency relations. To solve the above mentioned problems, Hochreiter et al. proposed the long short-term memory networks (LSTMs) that use a series of gating mechanisms to avoid simplification and compression of the gradient [10]. For the second problem, Miwa proposed tree LSTM [11]. The hidden layer unit in tree LSTM not only includes the previous sequence information but also integrates the information of the child nodes into the current node through the dependency relations. To solve cross-sentence n-ary relation extraction challenges. Peng et al. proposed the graph LSTM (Graph LSTM), which is a simplified version of tree LSTM because each node has a maximum of 2 incoming edges in the graph [5]. Song et al. proposed a graph-state LSTM model for cross-sentence n-ary relation extraction, which used a parallel state to model each word and enrich state values recurrently via message passing(GS GLSTM) [12]. Mandya et al. proposed a model of combining LSTM and a CNN for cross-sentence n-ary relation extraction. The proposed model brings together the properties of both LSTMs and CNNs, to simultaneously exploit long-range sequential information and capture most informative features (LSTM-CNN) [13].

Additionally, another type of graph neural network (GNN) has received considerable attention in natural language processing fields. GNN is a kind of neural network that can learn the attribute information of nodes and structure information of graphs [14]. Compared with RNNs alone, GNNs have certain advantages because GNN can capture the long-term dependencies of sentences through the constructed syntactic dependency. To solve the relation extraction task. Zhang et al. applied a graph convolutional network (GCN) over the pruned tree to extract relations[15]. Guo et al. proposed a soft-pruning approach that automatically learns how to selectively attend to the relevant important information [16], which used multihead attention applied on the dependency graph (AGGCN). The key idea behind the AGGCN is to use multihead attention to induce relations between nodes. In this paper, we use bidirectional long short-term memory networks (Bi-LSTM) to model cross-sentences as it can automatically and efficiently learn latent features from the input sequence. However, it is difficult to learn abundant latent features in the n-ary relations extraction. Therefore, we concatenate the Bi-LSTM layer with the multihead attention. The intuition behind the multihead attention is that applying the attention multiple time may learn more abundant features than single attention in the cross-sentence [17].

In addition, some relation extraction works have started to use a universal schema and knowledge representation learning to assist the model work [18–20]. In the universal schema, textual representations of entity pair and their relations are encoded into the same vector space as the canonical knowledge base relations. Knowledge representation learning is a method of transforming knowledge triplet data into low-dimensional vector space. The continuous representation of entities and relations obtained by this method retains the attribute information of the triples. TransE is a typical model of knowledge representation learning that uses a relation as the head entity to the tail entity translation operation [21]. For example, *e*_1_+*r*≈*e*_2_, where *e* is the entity and *r* is the relation. However, the TransE model has limitations when dealing with 1-N, N-1, and N-N complex relations. To solve this problem, Wang et al. proposed a TransH method in which an entity has different representations under different relations [22]. Lin et al. proposed a TransR method that ensures different relations have different semantic spaces [23]. For each triple, the entity should be projected into the corresponding relational space using the matrix, and then the translation relations from the head entity to the tail entity. For the heterogeneity and imbalance of entities in the knowledge base and the excessive matrix parameters in the TransR model, Ji et al. proposed a TransD method that optimized the TransR method [24]. However, knowledge representation learning has not yet been explored in the cross-sentence n-ary relation extraction.

In this paper, we propose a novel cross-sentence n-ary relation extraction method that utilizes multihead attention and knowledge representation learning from the knowledge graph (KG). The cross-sentence is relatively twice as long as the single sentence. A multihead attention mechanism directly draws the global dependencies of the inputs regardless of the length of the sentence. Knowledge representation learning makes use of entity and relation information from the KG to impose assistance while predicting the relation. Our method uses encoded context representation information obtained from multihead attention, along with embedded relation representation information, to improve cross-sentence n-ary relation extraction. Our contributions are summarized as follows:

- We propose a novel neural method that utilizes representation learning from the KG to learn prior knowledge in n-ary relation extraction.
- Our method first uses Bi-LSTM to model sentences and then uses the multihead attention to learn abundant latent features of the Bi-LSTM output.
- We conduct experiments on the cross-sentence n-ary relation extraction dataset and achieve state-of-the-art performance.

## Methods

In this section, we mainly introduce the components and architectures of the model.

### Knowledge representation learning

#### Construct knowledge graph

We use the Gene Drug Knowledge Database and the Clinical Interpretations of Variants in Cancer knowledge base to extract drug-gene and drug-mutation pairs [25]. There are five relations: “resistance or nonresponse”, “sensitivity”, “response”, “resistance” and “none” for the knowledge triples. Our KG is a directed graph $\mathcal {G} = (\mathcal {E,R,T})$, where $\mathcal {E}, \mathcal {R}$ and $\mathcal {T}$ indicate the sets of entities, relations and facts. Each triple $(h,r,t) \in \mathcal {T} $ indicates that there is a relation $r \in \mathcal {R}$ between $h \in \mathcal {E}$ and $t \in \mathcal {E}$. More generally, we can formalize two types of triples, such as (*e*_*d*_,*r*,*e*_*g*_) and (*e*_*d*_,*r*,*e*_*m*_). *e*_*d*_,*e*_*g*_,*e*_*m*_ and *r* indicate a drug entity, gene entity, mutation entity and a relation, respectively. After building the KG, we use the translation model to encode entities and relations uniformly. When performing relation extraction from sentence, we first obtain the identification of the entity from the sentence, and then use the identification to obtain the vector representation of the entity in the KG.

#### Translation model

**Fig. 1:**
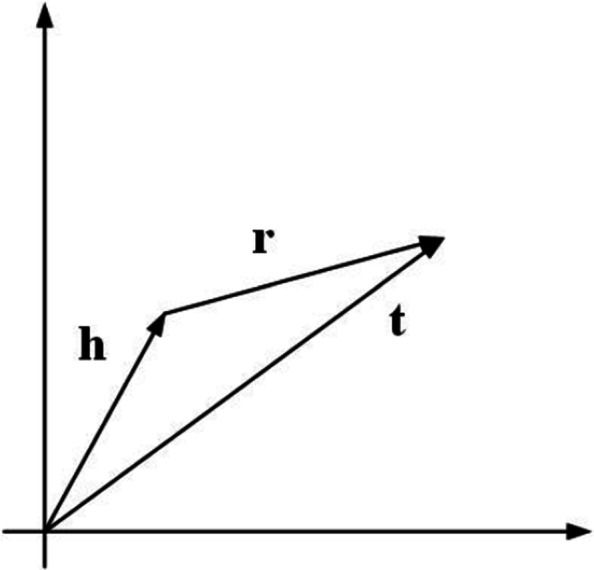
Simple illustration of TransE

The basic idea of a translation model is that the relations between two entities correspond to a translation between the embedded representations of two entities. In this paper, we mainly use the TransE, TransR, TransH and TransD methods to learn entity and relations representation [21–24, 26]. Taking the TransE method as an example, the relation in each triple instance is treated as a translation from the entity head to the entity tail by constantly adjusting *h*, *r*, and *t* (the vector of head, relation, and tail), making *h*+*r* as equal as possible to *t*; that is, *h*+*r*≈*t*. Figure 1 is a schematic diagram of the TransE model. we use the bold face **h**, **t** and **r** to indicate their low-dimensional vectors, respectively. $\mathbf {h}, \mathbf {t} \in \mathbb {R}^{k}, \mathbf {r} \in \mathbb {R}^{k}$, and *k* are the dimensions of both entities and relations. The loss function of TransE is defined as:

1 $$ \mathcal{L}= \sum_{(h, r, t) \in \mathcal{T}} \sum_{(h', r, t') \in \mathcal{T}'}[\gamma - \lVert \mathbf{h} + \mathbf{r} - \mathbf{t} \rVert + \lVert \mathbf{h}' + \mathbf{r} - \mathbf{t}' \rVert]^{+}  $$

*γ* is the margin hyperparameter, $\mathcal {T}'$ is a negative sampled triple set obtained by replacing **h** or **t**, and []+ is a positive value function. Motivated by the above method, we utilize a relation vector *r* to represent the features of the relation that links drug (*e*_*d*_), gene (*e*_*g*_) and mutation (*e*_*m*_), *r*≈*h*−*t*. In this paper, we explore whether the method of combining representation learning is more effective for cross-sentence n-ary relation extraction.

### The architecture of model

**Fig. 2:**
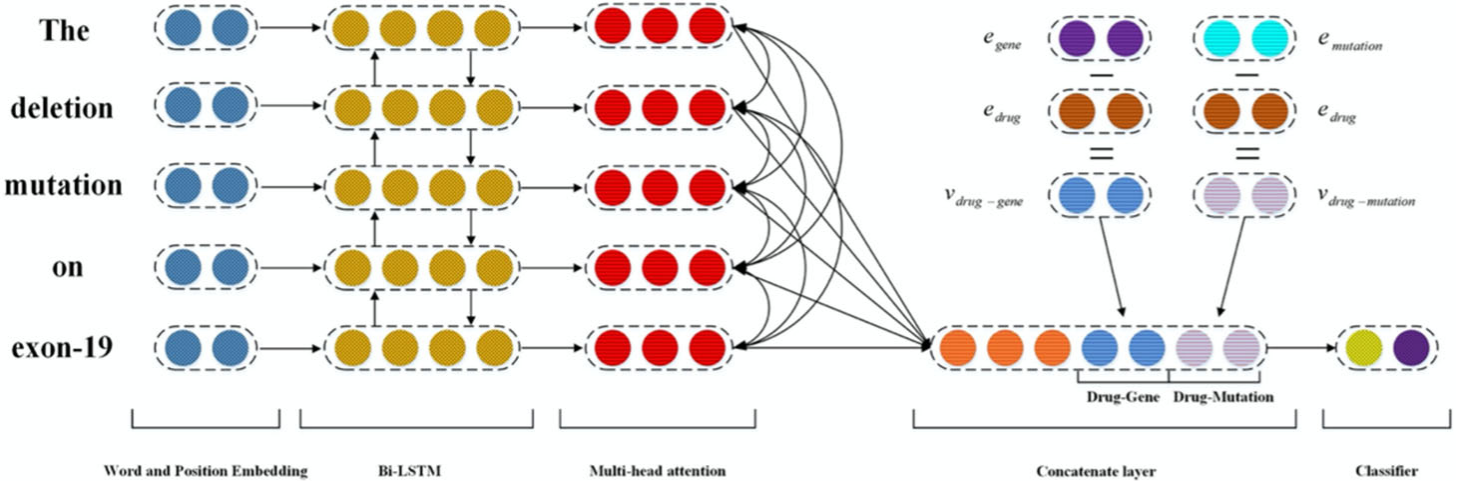
Overview of our model. The Bi-LSTM first encodes each word by concatenating word and position embeddings, followed the multihead attention directly draws the global dependencies of the Bi-LSTM output. Then, sentence embedding is concatenated with relation information, which comes from the KG. *e*_*drug*_,*e*_*gene*_ and *e*_*mutation*_ are the drug, gene and mutation entities, respectively. *v*_*drug*−*gene*_ and *v*_*drug*−*mutation*_ denote the different relation vectors. Finally, sentence representation with entity relation information is fed to a softmax classifier

Our model mainly includes four parts: the word and position embedding, the Bi-LSTM, the multihead attention and the concatenate layer. The overall architecture of our method is shown in Fig. 2.

### Word and position embedding

Converting words into low-dimensional vectors has been shown to effectively improve many natural language processing tasks. This paper uses Wikipedia and Web text pre-trained vectors to initialize the text embedding, and each word can be mapped to the corresponding feature vector through the pre-trained words^1^. In the relation extraction task, the position feature is essential [8]. Similarly, we also add position features in the cross-sentence n-ary relation extraction. It is calculated from the relative distance of the current word to the entity. Each word has three relative distances. For example, “*The deletion mutation on exon 19 of the****EGFR****gene was present in 16 patients, while The****L858E****point mutation on exon 21 was noted in 10. All patients were treated with****gefitinib****and showed a partial response.*”. The relative distances from the treated to the entity (EFGR), entity (L858E) and entity (gefitinib) are 22, 13 and -2, respectively. We randomly initialize the three-position embedding matrices and then convert the relative distances into vectors by lookup.

### Bidirectional long short-term layer

RNN is very suitable for processing sequence input and has been successfully applied to many NLP tasks. Compared with traditional RNN, LSTM uses a gating mechanism to mitigate gradient problems. In this paper, we use bidirectional long short-term memory networks (Bi-LSTM) to learn more contextual information. For a given sentence $X=(x_{1},x_{2}...,x_{n}), x \in \mathbb {R}^{k}$, *x* denotes the concatenating vector of the current word embedding and three position features, and the LSTM unit is calculated as follows:

2 $$\begin{array}{*{20}l} i& = \sigma(W_{xi}x_{t}+W_{hi}h_{t-1}+b_{i}) \end{array} $$

3 $$\begin{array}{*{20}l} f& = \sigma(W_{xf}x_{t}+W_{hf}h_{t-1}+b_{f}) \end{array} $$

4 $$\begin{array}{*{20}l} o& = \sigma(W_{xo}x_{t}+W_{ho}h_{t-1}+b_{o}) \end{array} $$

5 $$\begin{array}{*{20}l} g& = tanh(W_{xg}x_{t}+W_{hg}(i \odot h_{t-1})+b_{g}) \end{array} $$

6 $$\begin{array}{*{20}l} c_{t}& = f \odot c_{t-1} + i \odot g \end{array} $$

7 $$\begin{array}{*{20}l} h_{t}& = (1-f)\odot h_{t-1} +f\odot g \end{array} $$

*W*_∗_ and *b*_∗_ denote weight matrices and biases, *σ* is the sigmoid function and ⊙ is elementwise multiplication. At the time step *t*, each LSTM unit calculates the input word *x*_*t*_,*h*_*t*_ is the hidden state of the current time step *t*. The Bi-LSTM combines forward LSTM $\stackrel {\rightarrow }{h_{i}}$ and backward LSTM $\stackrel {\leftarrow }{h_{i}}$, which is denoted as $h^{bi-lstm}_{i}=[\stackrel {\rightarrow }{h_{i}};\stackrel {\leftarrow }{h_{i}}]$.

### Multihead attention

**Fig. 3:**
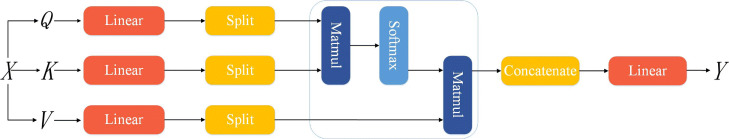
Overview of multihead attention

Although Bi-LSTM can effectively and automatically learn the latent features from the input sequences, it is difficult to learn abundant latent features in the n-ary relation extraction. The inspiration behind using the multihead attention mechanism is to learn the word dependence within the cross-sentence and capture the important information of the sentence. Figure 3 shows the calculation process of the multihead attention mechanism. Given $X \in \mathbb {R}^{n \times d}$ denoting the input vectors, multihead attention applies different linear projection functions to map the matrix *X* as the query $Q \in \mathbb {R}^{n \times d}$, key $K \in \mathbb {R}^{n \times d}$, and value $V \in \mathbb {R}^{n \times d}$. The multihead attention uses dot-product attention to compute the attention scores based on the following equation.

8 $$ attention(Q,K,V)=softmax(\frac{QK^{t}}{\sqrt{d}})V  $$

*d* denotes the number of hidden units. The key point of multihead attention is employing *h* parallel heads to focus on different parts of the value vector channels. For each head, we define the corresponding learning parameters, $W_{i}^{Q} \in \mathbb {R}^{n \times \frac {d}{h}}, W_{i}^{K} \in \mathbb {R}^{n \times \frac {d}{h}}, W_{i}^{V} \in \mathbb {R}^{n \times \frac {d}{h}}$, and the *i*-th head attention can be calculated as follows:

9 $$ M_{i}=Attention(QW_{i}^{Q},KW_{i}^{K},VW_{i}^{V})  $$

Splicing the *h* times scaled dot-product attention result, and then performing a linear transformation to obtain the value as the result of the multiheaded attention

10 $$ M=Concat(M_{1},...,M_{h})  $$

11 $$ Y=MW  $$

where $M \in \mathbb {R}^{n \times d}, W \in \mathbb {R}^{d \times d}$.

### Concatenate layer

Similar to many methods, we do not directly use the multihead attention output representation $\mathcal {B}$ but instead embed the embedding of each sentence with the translation relations of the corresponding entity obtained from the translation model [26].

12 $$ \check{\mathcal{B}}=[\mathcal{B};\mathcal{R}_{drug-gene};\mathcal{R}_{drug-mutation}]  $$

By using the translation model, we obtain a distributed representation of entities and relations. Furthermore, instead of directly using the training vector, we perform subtraction on the distributed representation of the two entities to obtain a corresponding relation vector representation.

13 $$ \mathcal{R}_{drug-gene}=\mathcal{E}_{gene}-\mathcal{E}_{drug}  $$

where $\mathcal {E} \in \mathbb {R}^{k} $. Similarly, for the drug-mutation relation,

14 $$ \mathcal{R}_{drug-mutation}=\mathcal{E}_{mutation}-\mathcal{E}_{drug}  $$

Finally, $\check {\mathcal {B}}$ is fed to the softmax classifier to obtain a probability distribution for each relation.

15 $$ p(y)=Softmax(W\cdot \check{\mathcal{B}}+b)  $$

## Results

### Dataset description

In order to build knowledge graph, we follow Peng to generate 137,469 drug-gene and 3,192 drug-mutation positive triples from the approximately one million biomedical fulltext articles [5]. The data we used were extracted by cross-sentence n-ary relation extraction, which extracts the drug-gene-mutation triples in the biomedical literature^2^. The data were constructed by 6,987 n-ary relation instances and 6,087 binary instances. Table 1 shows the statistics of the data. Most of the n-ary relation instances were contained in the cross-sentences, and the average number of sentences was two. There were 5 categories of relations: “resistance or nonresponse”, “sensitivity”, “response”, “resistance” and “none”. “None” indicates a negative instance, which is no reaction relations in the cooccurring entity. In the case of binary classifications (two categories), the labels of all positive case relations are denoted as “yes”, none denotes “no”, and with fine-grained classification, the data are labeled with five types of relations [12]. Five types of n-ary relation data examples are given below

- Sensitivity: *Exon 19 deletions and****L858R****mutations have shown similar in vitro sensitivity to gefitinib; however,****erlotinib****and gefitinib have shown different clinical efficacy depending on whether exon 19 deletions and L858R mutations are present. Despite these differences, both drugs have efficacy in patients with both of these mutations, and these differences do not influence treatment selection. As the number of clinical trials evaluating****EGFR****TKIs continues to increase, the number of patients eligible for pooled analyses such as this one increase.*

**Table 1:** Ternary and binary relation data statistic percentages indicate instances that contain multiple sentences

Data	Single	Cross	Positive	Cross-percentage
Ternary	2,301	4,956	3,462	70.1%
Binary	2,728	3,359	3,192	55.2%
- Resistance or nonresponse: *All of the patients had****EGFR****gene mutations in exon 19 (delE746-A750) or exon 21 (L858R) and received or were receiving gefitinib or****erlotinib****for treatment against advanced diseases at time of blood sampling. For analysis of EGFR gene mutations in exon 19 (delE746-A750) or exon 21 (L858R), the peptic nucleic acid locked nucleic acid (PNA-LNA) polymerase chain reaction (PCR) clamp method was adopted using protocols described previously. The EGFR****T790M****mutation was examined in cell-free DNA obtained from the plasma of patients since no biopsy specimens for DNA analysis could be obtained because of the difficult accessibility of tumors during or after EGFR-TKI treatment.*
- Response: *The appearance of a second mutation represents a mechanism of resistance. In fact, the authors demonstrate that the insertion of****T790M****into test cells renders them resistant to gefitinib in vitro. They also found that when test cells transfected with both mutations are treated with other****EGFR****inhibitors, such as AG1478,****cetuximab***, *erlotinib or CL-387,785, no objective response is obtained using the first three agents, while the fourth is effective.*
- Resistance: *This analysis included****F1174L***, *from the SH-SY5Y neuroblastoma cell line, as well as a number of additional previously uncharacterized ALK mutations, and looked at their transformation potential. However, this work did not examine whether the various****ALK****mutants were able to respond to activation by external ligand or agonist antibodies or examine their sensitivity to treatment with****crizotinib***.
- None: *This shows how vemurafenib can be beneficial for tumors of one molecular phenotype* (***V600E****mutant) but potentially adverse for another (HRAS/NRAS mutant). Molecular therapeutics in melanomas are not restricted to treatments directed at the****MAPK****pathway. In a recent Phase II study of 43 patients with metastatic melanoma with KIT aberrations (mutation or amplification) treated with****imatinib***, *an overall response rate of 23.3% was observed.*:

### Parameters setting

In this paper, we use the average accuracy of the five-fold cross validation to verify the performance of the model. In our experiments, our model is based on TensorFlow as the back-end computational framework [27]. We use cross-entropy as the loss function. To prevent overfitting the model during training, dropout techniques are used in different layers of the model [28]. Hyper parameters were set based on preliminary experiments on a small development dataset. The parameters used are shown in Table 2. The vector initializes the 200-dimensional word vector through GloVe. while the word vector is obtained through Wikipedia and web text [29], the number of hidden units in the LSTM is 200, the minimum batch is 6, the learning rate of Adam is 0.001 [30], and the number of epochs is 10, the number of heads is 4. We use TransR as the main translation model in the experiment. The final experimental results select the best experimental model on the validation set and use the test set for verification. Like Song, we randomly select 200 instances from the training set as the verification set [12].

**Table 2:** Parameters design

Parameter name	Value
Word embedding dimension	200
Subrelation embedding dimension	50
Position embedding dimension	50
Recurrent dropout for Bi-LSTM	0.5
GCN dropout probability	0.5
Batch size	6
Adam-learning rate	0.001
Hidden state dimension of Bi-LSTM	200
Hidden state dimension of multihead	400
Multihead attention head	4

### Experimental results

“Ternary” and “binary” denote ternary drug-gene-mutation (entity triples) interactions and binary drug-mutation (entity pairs) interactions, respectively. “Single” represents experiments only on instances within single sentences, while “Cross” represents experiments on all instances.

### Compare with baseline methods

To evaluate the effectiveness of our proposed method in the cross-sentence n-ary relation extraction task, we consider feature-based, hybrid, and graph models as baselines. For ternary relation extraction (first two columns in Table 3), our multihead attention achieves accuracies of 81.5 and 87.1, respectively. In all instances of cross-sentences, our multihead attention achieves the same performance as the state-of-the-art AGGCN and outperforms other baselines. Compared with the graph-based method, our method does not require the process of text-to-graph conversion and enables a higher accuracy. AGGCN used the combination of a densely connected layer and an attention guided layer to learn representations of graphs [16]. Compared with AGGCN, our method has a simpler architecture and enables the same accuracy. We notice that our method achieves better accuracy than all GCN models, which further demonstrates its ability to learn global dependencies. We also report accuracies only on instances within single sentences (column Single in Table 3), which exhibited broadly similar trends. Note that all methods except AGGCN drop performance when evaluated only on single-sentence relations, which are more challenging. The reason for this phenomenon is that the training data is relatively small in the single sentence, as only 30% of instances are within a single sentence. Another possible reason is that the context information provided in a single sentence is insufficient.

**Table 3:** Average test accuracy in five-fold cross validation of the proposed model and state-of-the-art methods on cross-sentence n-ary relation extraction. “-” denotes that the value is not provided herein. Full Parametrization (FULL) denote as each edge label is associated with a 2D weight matrix to be tuned in training. Type Embedding (EMBED) denote as each edge label to an embedding vector. K in the GCN models means that the preprocessed pruned trees include tokens up to distance K away from the dependency path in the lowest common ancestor subtree. *: significant at *p*<0.005

Method	Ternary		Binary	
	Single	Cross	Single	Cross
Feature-based [31]	74.7	77.7	73.9	75.2
LSTM-CNN [13]	79.6	82.9	85.8	88.5
Graph LSTM-EMBED [5]	76.5	80.6	74.3	76.5
Graph LSTM-FULL [5]	77.9	80.7	75.6	76.7
Graph LSTM MULTITASK [5]	-	82.0	-	78.5
GS GLSTM [12]	82.3	85.5	85.4	85.6
GCN (K=0) [15]	85.6	85.8	82.8	82.7
AGGCN [16]	87.1	87.0	85.2	85.6
Bi-LSTM	80.8	85.9	88.6	89.3
GNN	83.0	86.6	88.7	88.6
Multihead attention	81.5	87.1	89.7*	90.6*
With KG	87.3*	91.9*	-	-

These results also suggest that compared to previous feature based method which use a statistical method with the features derived from shortest paths between all entity pairs, variant graph LSTMs (Graph LSTM, GS GLSTM) are able to extract valuable information from the underlying tree structure. Compared with variant graph LSTMs, GNN based methods (GCN, AGGCN) can learn a more expressive representation through graph convolutions. The hybrid neural network method combines the advantages of LSTM and CNN and also achieves a considerable result.

We extend the multihead attention method with a translation model to capture the relation representations, which are subsequently fed into softmax layers. Using all instances (the cross column in Table 3), our method shows the highest test accuracy among all methods, which is 4.8% higher than our baseline^3^. Through experimental analysis, we observe that the multihead attention mechanism concatenate knowledge graph can detect more positive examples.

### Fine-grained classification

In this paper, we have carried out multi-class classification experiments on cross-sentence n-ary relation extraction. For the multi-class relation extraction task, we also report the macro-averaged F1 score. Table 4 shows the accuracy and F1 score of the multi-class relation extraction. In terms of accuracy and F1 score, our method leads current state-of-the-art methods by 7.5% and 10.7%, respectively. In addition, we observe that after concatenating the KG, our method can detect more “resistance or non-response” and “sensitivity” categories, but instead detect the number of “none” category relations begin to decrease. This phenomenon is also attributed to prior knowledge which to provide valuable information for sentences.

**Table 4:** Average test accuracies and F1 for multi-class relation extraction with all instances

Method	Multi-class			
	Ternary accuracy	Ternary F1	Binary accuracy	Binary F1
GS GLSTM [12]	82.3	76.1	82.1	75.8
GCN (K=0) [15]	78.1	74.6	73.1	70.2
AGGCN [16]	79.7	75.5	77.5	73.5
Multihead attention	86.8	84.3	91.6	88.8
With KG	89.8*	86.8*	-	-

#### Multihead attention results

We assessed the effectiveness of multihead attention in n-ary relation extraction. In this experiment, all models used a multihead attention mechanism and the combination of word and position embedding as input representations. To verify the influence of the different heads, we randomly selected several heads from {2,4,8}. Table 5 shows the results. Multihead attention can be combined with important features from different heads to represent a comprehensive feature. We notice that when the number of heads is set to 2 or 8, the performance will drop off. Overall, multihead attention achieved the highest accuracy of 87.1 when the number of heads was 4.

**Table 5:** Average test accuracies in five-fold validation for different numbers of head attention

Method	Ternary		Binary	
	Single	Cross	Single	Cross
2-head	81.2	85.8	91.4	89.1
4-head	81.5*	87.1*	89.7	90.6
8-head	81.2	86.7	91.4	90.7

#### Performance comparison of basic models

In Table 3, we find that GNN is better than Bi-LSTM, except that it performs slightly worse in the cross-sentence binary relation extraction. Compared with Bi-LSTM, GNN can learn effective information, which fully indicates that GNN can capture effective information by using the document graph. We also see that the overall performance of the multihead attention mechanism exceeds Bi-LSTM, which fully demonstrates that the multihead attention mechanism can learn global dependency information, whether it is a long or a short sequence. Compared with Bi-LSTM, the multihead attention mechanism has been improved in identifying the number of positive and negative examples, especially for relatively long sequences. Additionally, we observed that the performance of the multihead attention mechanism also exceeded that of the GNN. This phenomenon shows that the multiattention mechanism network can learn more effective information than the GNN. In terms of input features, GNN not only needs word and position embedding but also requires a document graph. In the process of converting text from sequence to graph, not only does it require considerable time, but the parsing document may also have noise data. The multihead attention mechanism does not require any external processing technology, and it can achieve good performance, which shows that the multihead attention mechanism is more suitable for cross-sentence n-ary relation extraction.

#### The impact of position embedding on the model performance

From Table 6, we can see that position embedding plays an essential role in binary relation extraction. After adding the position embedding, the accuracy increases by 6.1% and 6.2%. Similarly, adding position embedding can greatly improve the performance of n-ary relation extraction. Position features are useful for multihead attention models by providing coded information on the location of word entities within a useful text range, which helps achieve greater accuracy. Without position embedding, the multihead attention only achieves an accuracy of 78.7 on the cross-sentence n-ary relation. When using the position embedding approach, the accuracy improves to 87.1.

**Table 6:** Average test accuracies in five-fold validation for the effect of position embedding

Method	Ternary		Binary	
	Single	Cross	Single	Cross
word	76.0	78.7	83.6	84.4
word+position	81.5*	87.1*	89.7*	90.6*

### The effect of representation learning

We further study the effects of several knowledge representation learning methods on n-ary relation extraction. In the experiment, we used four representation learning methods, namely, TransE, TransR, TransH and TransD. In this paper, we use the multihead attention mechanism as a baseline model that does not include representation learning. Here, we do not provide the performance of representation learning in binary relation extraction since it indicates that representation learning has obtained the category of binary relations. Therefore, it is not appropriate to add the binary relation representation to the text. Table 7 shows the results. The combination of the multiattention mechanism and the representation learning performance is superior to that without representation learning, which indicates that the knowledge representation can reveal the semantic links of entities and relations. TransE is simple to model 1-N, N-1 and N-N relations, and entities and relations are all modeled in the same union space; however, entities and relations are different types of data, and not all are suitable on a single space. Instead, the other three models map the relations to another space. Through the analysis of the experimental results, we find that the number of positive and negative examples identified by TransR has increased compared to TransE and TransD. Compared with TransH, the number of negative examples identified by TransR is almost the same, but the number of positive examples has been greatly improved. Overall, the best performance in cross-sentence n-ary relation extraction is TransR, which translates entities and relations in separate entity and relation spaces, ensuring the diversity of information. In addition, we explored the impact of the representation of the two subrelations on the overall model. Compared with the no representation learning method, using any subrelation representation has a beneficial impact. The experimental results are shown in Table 8. Of course, the model learns that two types of relation representations will further improve the performance. Overall, we observed that compared with models without KG, models which integrate with different type KG can detect more positive instances.

**Table 7:** Average test accuracies in five-fold validation for knowledge representation learning

Method	Ternary	
	Single	Cross
TransE	83.9	90.8
TransD	85.8	90.9
TransH	86.5	91.2
TransR	87.3*	91.9*

**Table 8:** Average test accuracies in five-fold validation for the different subrelations

Method	Ternary	
	Single	Cross
With drug-gene relation	83.1	89.5
With drug-mutation relation	82.7	89.5
With two relation	87.3*	91.9*

### Sentence length analysis

**Fig. 4:**
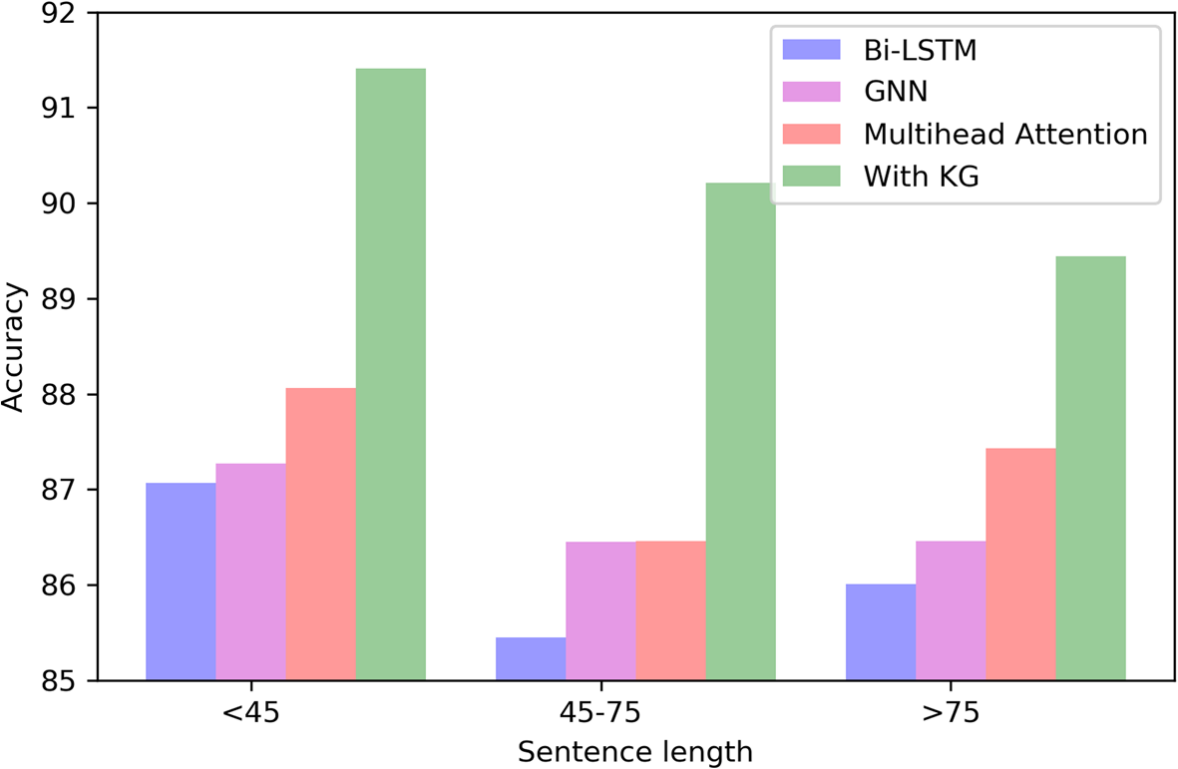
Test set performances on different sentence lengths

Figure 4 shows the accuracy of the four models under different sentence lengths. We divide the length of the sentence into three ranges: 0-45, 45-75, and 75-. We can see from Fig. 4 that the multihead attention mechanism performs best at any length except with the KG model. Compared with GNN, the advantage in the range of 45-75 is not particularly obvious. The possible reason is that the semantic parsing of the short sentence is more accurate, and GNN can learn more effective knowledge in short sentences. Overall, the performance of Bi-LSTM is relatively poor. Both GNN and the multihead attention mechanism can learn the internal structure of the sentence. In addition, we observe that compare with baseline models, multihead attention with KG has the best performance at any length, and the rate of accuracy increase is relatively large. As a result, it is inferred from the experiment that the performance of the multihead attention with KG is the best regardless of the length of the sentence.

### Error analysis

Tables 9 and 10 show the multi-class result confusion matrix on the same fold set. The x-axis is the predicted label by our method, and the y-axis is the gold standard label. From the results in Tables 9 and 10, we can observe that compared with the multihead attention, although the number of correct “none” relations identified by the model combined with KG is decreasing, the number of other four types of relations can be correctly identified are increasing, especially the number of “resistance or nonresponse” relations has increased significantly, from 168 to 227. In Table 10, we can see that the major challenge is “none” relation being mistaken for relations and vice versa. In addition, we perform error analysis on some sample prediction errors and give some examples. The drug, gene and mutation entities are in bold. For example, “*There are several promising agents for patients with activating EGFR mutations who experience disease progression of an EGFR tyrosine kinase inhibitor and have a****T790M****resistance mutation, and multiple clinical trials will be available. Trials investigating adjuvant erlotinib in EGFR mutant NSCLC and comparing erlotinib to****erlotinib****plus bevacizumab in metastatic****EGFR****mutant NSCLC are ongoing.*”. We found that predictive error instances are caused by the presence of multiple entities. Duplicate entities are more likely to behave as noise. Therefore, an improved strategy is needed to handle this situation. Replacing a duplicate entity with a specific tag may be one method for handling this situation. In another case, the three entities do not have an n-ary relation in the sentence. However, in the KG, some of the pairs may have a relation, which makes most samples nonrelated, but the model is mistaken for a relation. For example, *At least 10 other activating mutations (less common single amino acid substitutions such as* “***D761Y***, *L747S, and T854A) have been reported within the kinase domain, and the novel E884K mutation has been associated with resistance to gefitinib and****erlotinib***. *Balak et al. noted that given the proportion of patients with acquired resistance, whose tumors contain T790M, malignant cells remain dependent on mutant****EGFR****for survival in at least half of patients*”. In the KG, entities **erlotinib** and **D761Y** have a “none” relation, but **erlotinib** and **EGFR** have a “response” relation. In this case, our model failed to identify the nonrelation in the document. In future plans, more efforts should be made to explore how to better utilize the KG.

**Table 9:** Multi-class confusion matrix for multihead attention on the one fold set

Gold	none	resistance or	sensitivity	response	resistance
		non-response			
none	721	7	10	16	32
resistance or	74	168	0	4	2
non-response					
sensitivity	22	0	57	0	13
response	16	0	0	55	0
resistance	49	1	0	0	302

**Table 10:** Multi-class confusion matrix for multihead attention with KG on the one fold set

Gold	none	resistance or	sensitivity	response	resistance
		non-response			
none	701	11	17	5	42
resistance or	17	227	0	3	1
non-response					
sensitivity	13	0	78	0	1
response	12	0	0	59	0
resistance	43	0	0	0	309

## Conclusion

We explored a novel method for cross-sentence n-ary relation extraction. Unlike previous approaches, our methods operate directly on the sequence and learn to model the internal structure of sentences. In addition, we introduce the knowledge representations learned from the KG into the cross-sentence n-ary relation extraction. Experiments based on knowledge representation learning show that entities and relations can be extracted in the KG, and coding this knowledge can provide consistent benefits. Experimental results show that combining knowledge representation learning achieves state-of-the-art results on cross-sentence n-ary relation extraction.

In the future, we plan to work with healthcare professionals to apply our approach to clinical decision making. In particular, automatically extracted facts can serve as candidates for manual curation. However, in this paper, we only construct a small KG for representation learning. The relations we learn are only the relations between drug-gene, drug-mutation, and many biomedical binary relations that we have not yet applied. For example, the relations between gene-disease and drug-disease. We can use other binary relations to build a larger KG for rich knowledge representation learning.

## Abbreviations

DDI: Drug-drug interaction
PPI: Protein-protein interaction
CPI: Chemical-protein interaction
NLP: Natural language processing
KG: Knowledge graph
CNN: Convolutional neural network
RNN: Recurrent neural network
LSTM: Long short-term memory
Bi-LSTM: Bidirectional long short-term memory
GNN: Graph neural network

## Footnotes

1 http://nlp.stanford.edu/projects/glove/

2 The dataset is available at http://hanover.azurewebsites.net.

3 *p*<0.005 using t-test. The significance tests are performed against the best performing baseline. For the remaining of this paper, we use the same measure for statistical significance.
